# Supplementary material for: Rapid re-identification of human samples using portable DNA sequencing
Source: eLife. 2017 Nov 28;6:e27798. doi: 10.7554/eLife.27798 (PMC5705215; doi:10.7554/eLife.27798)
Supplement: Supplementary file 1. — Run statistics for the MinION sketch experiments. [file elife-27798-supp1.docx]

**Supplementary file – Tables**

Rapid DNA Re-Identification for Cell Line Authentication and Forensics

Sophie Zaaijer^1,2^**^+^**, Assaf Gordon^2^, Daniel Speyer^1,2^, Robert Piccone^3^ , Simon Cornelis Groen^4^, Yaniv Erlich^1,2,5+^

^1^ Department of Computer Science, Fu Foundation School of Engineering, Columbia University, New York, NY, USA.

^2^ New York Genome Center, New York, NY, USA.

^3^ Data Science Institute, Columbia University, New York, NY, USA.

^4^ Department of Biology, Center for Genomics and Systems Biology, New York University, New York, NY, USA.

^5^ Center for Computational Biology and Bioinformatics (C2B2), Department of Systems Biology, Columbia University, New York, NY, USA.

[agordon@nygenome.org](mailto:agordon@nygenome.org), [dspeyer@nygenome.org](mailto:dspeyer@nygenome.org), [robert.piccone@gmail.com](mailto:robert.piccone@gmail.com), sc.groen@nyu.edu

^+^ Corresponding authors: [sophie@cornell.edu](mailto:sophie@cornell.edu) , yerlich@nygenome.org

**Supplementary file 1A**

**Experimental Summary**

| **Exp #** | **Sample** | **Source** | **chemistry** | **ONT Kit** | | **DNA processing*** | **Operation+** | **Figure** |
| --- | --- | --- | --- | --- | --- | --- | --- | --- |
| 1 | NA12890 | gDNA | R7 | 2D | SQK-MAP006 | Standard lab | Students | Fig 3 |
| 2 | YE001 | Spit Kit | R7 | 2D | SQK-MAP006 | Standard lab | Students | Fig 2B, Fig S2.1 |
| 3 | SZ001 | Spit Kit | R9 | 2D | SQK-NSK007 | Standard Lab | Students | Fig 2D, Fig S4.1 |
| 4 | JP001 | Spit Kit | R9 | 2D | SQK-NSK007 EXP-NBD002 | Standard lab | In house | Fig 2C, Fig3B, Fig S3 |
| 5 | THP1 | Cell culture | R9 | 2D | SQK-NSK007 EXP-NBD002 | Standard lab | In house | Fig 4, Fig S4.1, Fig5 |
| 6 | SZ001 | Spit kit | R9 | 1D | SQK-RAD001 | Standard lab | In house | Fig 6B |

^*^ DNA processing indicates the type of equipment used for most of the library preparation steps.

^+^ Operation denotes the group that operated the MinION for the sequencing experiment. Students: Columbia University undergraduate and Masters students as part of the course “Ubiquitous Genomics” 2015 (Zaaijer et al., 2016). In house: one of the authors (S.Z).

**Supplementary file 1B**

|  | **NA12890**  **2D** | **YE001**  **2D** |
| --- | --- | --- |
|  | **Sequencing yield** | |
| Passed bases (#) | 17,675,127 | 48,451,196 |
| Passed reads (#) | 2,272 | 10,067 |
| Read length average (bp) | 7,779 | 4,812 |
| Unique aligned reads (#) | 1,451 | 7,808 |
| Aligned bases (#) | 27,810 | 112,988 |
| Avg. read error rate (%) | 9.6 | 7.4% |
|  | **Matching details** | |
| **#SNPs to positive identification*** | **195** | **110** |
| Match homozygous genotype | 54 | 76 |
| Homozygous mismatch | 10 | 2 |
| Match heterozygous genotype | 131 | 7 |
| Time to positive identification (min.) | 13min | 13min |

*positive identification was defined as 99.9% for 2D experiments

**Supplementary file 1C**

|  | **SZ001**  **2D** | **JP001**  **2D** |
| --- | --- | --- |
|  | **Sequencing yield** | |
| Passed bases (#) | 33,216,820 | 21,369,107 |
| Passed reads (#) | 8,610 | 7,425 |
| Read length average (bp) | 3,857 | 2,878 |
| Unique aligned reads (#) | 6,127 | 5,783 |
| Aligned bases (#) | 98,504 | 67,402 |
| Avg. read error rate (%) | 3.8 | 3.4 |
|  | **Matching details** | |
| **#SNPs to positive identification*** | **98** | **134** |
| Match homozygous genotype | 66 | 88 |
| Homozygous mismatch | 3 | 4 |
| Match heterozygous genotype | 29 | 42 |
| Time to positive identification (min.) | 11.4min | 4.7min |

*positive identification was defined as 99.9% for 2D experiments

**Supplementary file 1D**

|  | **THP1**  **pure** | **THP1**  **contaminated** |
| --- | --- | --- |
|  | **Sequencing yield** | |
| Passed bases (#) | 11,721,501 | 31,283,238 |
| Passed reads (#) | 3,823 | 9,555 |
| Read length average (bp) | 3,066 | 3,274 |
| Unique aligned reads (#) | 3,594 | 8,991 |
| Aligned bases (#) | 38,135 | 98,705 |
| Avg. read error rate (%) | 5.24 | 5.20 |
|  | **Matching details** | |
| **#SNPs to positive identification*** | **91** |  |
| Match homozygous genotype | 72 |  |
| Homozygous mismatch | 1 |  |
| Match heterozygous genotype | 18 |  |
| Time to positive identification (min.) | 3min |  |

*positive identification was defined as 99.9% for 2D experiments

**Supplementary file 1E**

|  | **Rapid Kit**  **In LAB** | |
| --- | --- | --- |
|  | **Pass + fail** | **Passed only** |
|  | **Sequencing yield** | |
| Avg. base calling quality | 5.9 | 7.8 |
| All bases (#) | 209,580,567 | 8,367,648 |
| Reads (#) | 96,988 | 3345 |
| Read length average (bp) | 2161 | 2501 |
| Aligned reads (#) | 68,475 | 3207 |
| Aligned bases (#) | 111,481 | 26178 |
| Avg. read error rate (%) | 20 | 10.3 |
|  | **Matching details** | |
| **#SNPs to positive identification*** | **471** | **239** |
| Match homozygous genotype | 285 | 147 |
| Homozygous mismatch | 46 | 18 |
| Match heterozygous genotype | 140 | 74 |
| Time |  | 2.3 hrs |

*Positive identification was defined as 99.9% unless otherwise indicated
